# Quantitative Determination of ABT-925 in Human Plasma by On-Line SPE and LC-MS/MS: Validation and Sample Analysis in Phase II Studies

**DOI:** 10.3390/pharmaceutics2020171

**Published:** 2010-05-04

**Authors:** Katty Wan, Matthew Rieser, Tawakol El-Shourbagy

**Affiliations:** Abbott/100 Abbott Park Road, Abbott Park, IL 60064, USA; E-Mails: matthew.j.rieser@abbott.com (M.R.); tawakol.a.elshourbagy@abbott.com (T.E.)

**Keywords:** On-line SPE, LC-MS/MS, multiplexing for high throughput

## Abstract

A fully automated 96-well On-Line Solid Phase Extraction (SPE) followed by High Performance Liquid Chromatography (HPLC)-Tandem Mass Spectrometric (MS/MS) method for the determination of ABT-925 (2-{3-[4-(2-tert-Butyl-6-trifluoromethyl-pyrimidin-4-yl)-piperazin-1-yl)-propyl-sulfanyl}-3H-pyrimidin-4-one fumarate) in human plasma was developed, validated and utilized in Phase II clinical studies. 50 µL of plasma sample was fortified with internal standard (IS, d8-ABT-925) and extracted on-line with Cohesive Turbo Flow Cyclone P HTLC column. The chromatographic separation was performed on Aquasil C18 (3 μm 50 × 3 mm) HPLC column with a mobile phase consisting of 50/50/0.1 (v/v/v) ACN/H2O/formic acid. The mass spectrometric measurement was conducted under positive ion mode using multiple reaction monitoring (MRM) of *m/z* 457.4 → 329.4 for analyte and *m/z* 465.5 → 337.5 for IS. The peak area ratio (analyte/IS) was used to quantitate ABT-925. A dynamic range of 0.0102 μg/mL to 5.24 μg/mL was established after the validation. The validated method was then used for two Phase II studies. To demonstrate the method reproducibility, approximately 10% of the incurred samples from one study were repeated in singlet. The repeated values were compared to the initial values. All repeated values agreed within ±15% of the mean values.

## 1. Introduction

Pharmacological antagonism of central dopamine receptors has been demonstrated to be effective in treating symptoms of psychotic disorders. The conventional drug therapy of schizophrenia has used neuroleptics, which pharmacologically act as antagonists at the dopamine D2 receptor. However, medical treatment of dopamine related disorders is often limited by neurological and endocrine adverse events as a result of binding to various dopamine or other related monoamine receptors [1]. Unlike D2 receptors, which are extensively expressed in the limbic and the nigro-striatal regions of the brain, dopamine D3 receptors are more densely expressed only in the limbic region [2]. Therefore, selective blockade of dopamine D3 receptors represents an attractive approach to the therapy of psychotic disorders. ABT-925 is a substituted piperazinyl-pyrimidone derivative. Radioligand binding studies characterize ABT-925 as a selective D3 dopamine receptor antagonist with high affinity and selectivity for D3 receptors. 

To support pharmacokinetic evaluations in the D3 receptor occupancy study in humans via positron emission tomography (PET) scans, it is necessary to develop and validate a robust analytical method with adequate selectivity, accuracy and precision, and sensitivity. Historically, the method was developed in-house as a HPLC-UV method in which 1000 µL of plasma was needed to achieve 0.017 μg/mL lower limit of quantitation (LLOQ). The analytical run time was relatively long (~20 min) too. 

In order to increase sample throughput, a tandem mass spectrometric method was developed and validated using a fully automated 96-well on-line SPE method followed by LC-MS/MS measurements. The method used only 50 µL of plasma and the run time (including on-line SPE) was less than 5 min. When multiplexed (2×) HPLC platform was used, less than 3 min per sample throughput was achieved. Using On-line extraction and automation allowed for fewer steps, minimum human intervention and resulted in less errors and run failures. Incurred sample re-analysis demonstrated the excellent reproducibility of the method. 

## 2. Experimental Section

### 2.1. Chemicals and materials

**Figure 1:**
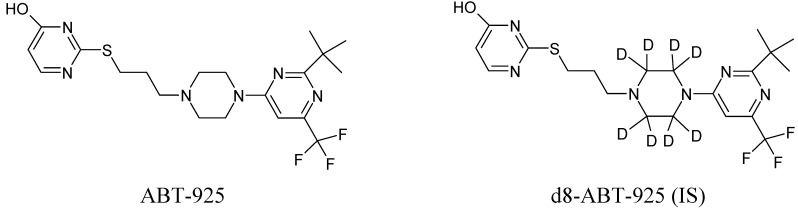
Chemical Structures of ABT-925 and its stable label internal standard d8-ABT-925.

ABT-925 (potency 79.2%) and its stable label internal standard (IS, d8-ABT-925) were provided by Abbott (Abbort Park, IL, USA). Figure 1 shows the chemical structures for both analytes. Formic acid (>98%), methanol (MeOH, HPLC grade) and acetonitrile (ACN, HPLC grade) were obtained from EMD. Ammonium hydroxide (28.0–30.0%) was obtained from J.T. Baker (Phillipsburg, NJ, USA). Ultrapure water was obtained from a Milli-Q Plus water purification system (Millipore, Bedford, MA, USA). Blank plasma with potassium EDTA as anticoagulant was purchased from Biological Specialty (Colmar, PA, USA).

### 2.2. Preparation of stock and working solutions

Due to the highly hygroscopic nature of ABT-925, a Karl Fischer test to determine the water content must be perform on the day of analyte weighing. 

Solid analyte and IS were accurately weighed into volumetric flasks using an analytical balance. They were then diluted to volume with 20% methanol. The stock solutions for spiking the calibration standards and quality control samples were prepared from separate weighing at approximately 500 µg/mL. IS working solution at approximately 50 ng/mL was diluted from IS stock solution with acetonitrile. All stock solutions and working solution were stored in glass vials in a refrigerator at about 2–8 ºC. 

### 2.3. Preparation of calibration standards and QC samples in human plasma

Small volumes (<5% of total preparation volume) of stock solutions were spiked initially with calibrated gas tight syringes into human plasma for the preparation of Calibration standard (STD) and quality control (QC) samples. STDs and QCs of lower concentrations were then prepared in human plasma using class “A” volumetric glassware by serial dilutions. The theoretical concentrations of STDs were 5.24, 2.62, 1.31, 0.655, 0.327, 0.164, 0.0818, 0.0409, 0.0205 and 0.0102 µg/mL. The theoretical concentrations of QCs were 4.16, 0.999, 0.160 and 0.0256 µg/mL. STDs and QCs were aliquoted into polypropylene cryogenic vials and kept frozen at -20 ºC until use.

### 2.4. Sample preparation

First, a volume of 250 μL of IS working solution was added to each appropriate well of a 96-well plate. Then 50 μL of STDs, QCs, Blank plasma, and Unknowns (if applicable) were added to the appropriate wells. The plate was covered with pierce plate cover and vortexed on low/medium speed for approximately 2 min. Samples in the plate were then centrifuge for about 10 min at approximately 3400 rpm to sediment the precipitation. Finally the plate was transferred into a cooled autosampler (temperature set point at 10 ºC) and 30 μL of samples from each well were injected, extracted on-line by SPE, and analyze using LC-MS/MS.

### 2.5. On-line solid phase extraction

**Figure 2:**
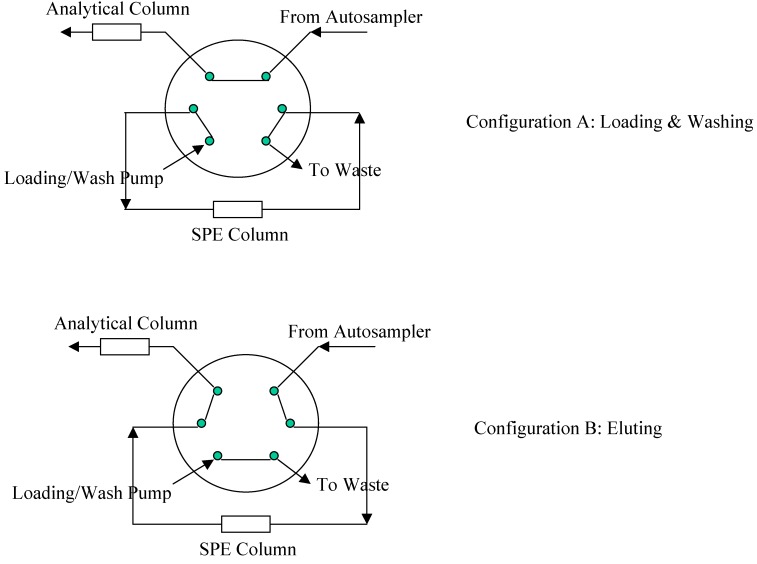
Valve Configuration for On-line SPE.

Analyte of interest was extracted from normal human plasma using on-line SPE technique. TurboFlow Cyclone P HTLC column (1 × 50 mm) was purchased from Thermo Scientific Inc. (Franklin, MA, USA). A CTC HTS PAL autosampler (Leap Technology, Carrboro, NC, USA) was used to perform the SPE extraction and the HPLC separation. Figure 2 illustrates the valve setup for on-line SPE. A column-switching valve was used to direct the flow from the extraction column either to waste (offline, Configuration A) or to the analytical column (online, Configuration B). Initially, loading solution (0.1% formic acid) flowed at 4.0 mL/min through the extraction column to waste for approximately 1 min (Configuration A). The column-switching valve then switched so that mobile phase from the analytical pump, eluted analyte from the extraction column onto the analytical column (Configuration B). After elution, the column-switching valve switched back to its original position (Configuration A) to wash (with 0.1% NH_4_OH followed by 100% ACN) and re-equilibrate (with 0.1% formic acid) the extraction column at 4.0 mL/min flow rate. With approximately 2 min remaining in the run, the mass spectrometer began to acquire data and a second column-switching valve (not shown in Figure 2) directed the analytical column flow from waste into the mass spectrometer. At the end of acquisition, the system was ready for the next injection. 

### 2.6. Chromatographic conditions

**Figure 3:**
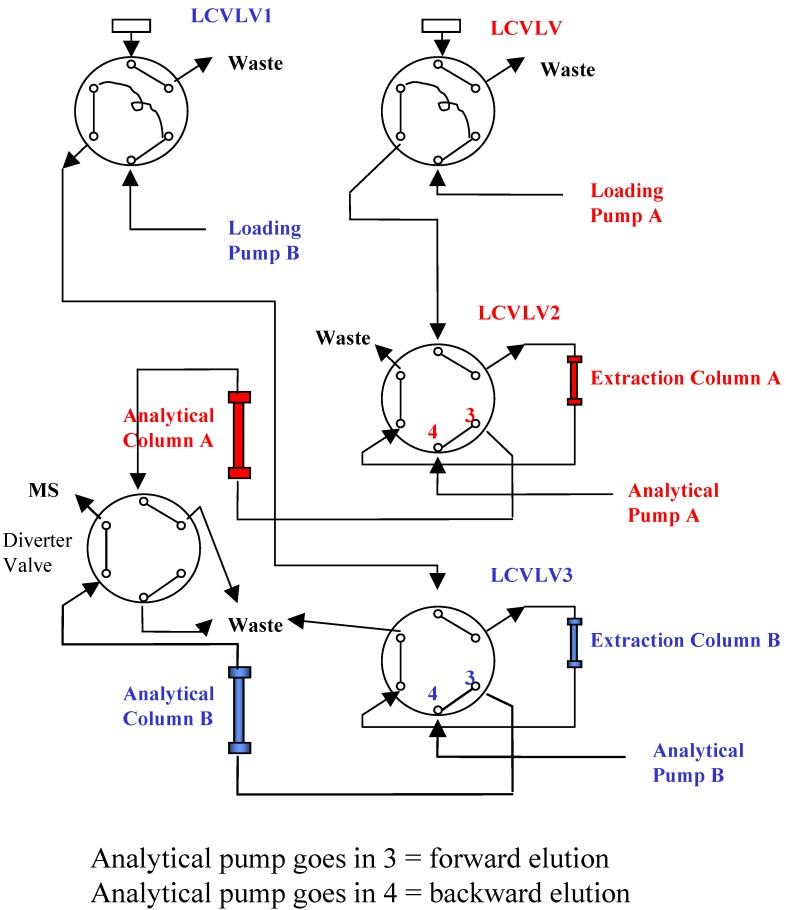
Valve Configuration for Paralleled On-line SPE.

**Figure 4:**
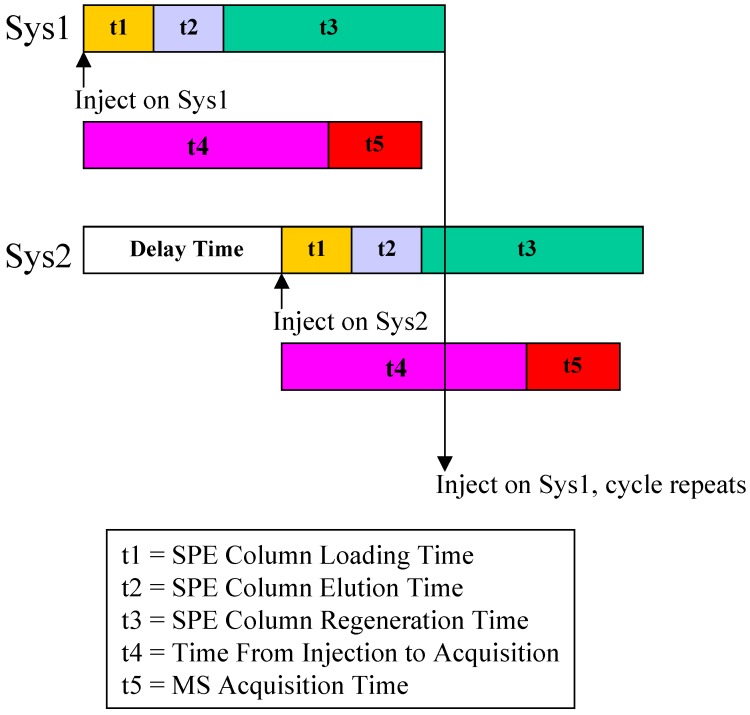
Timing Configuration for Paralleled On-line SPE.

Chromatographic separation of analyte and IS was achieved by using Thermo Aquasil C18 3 µm 50 × 3 mm column at room temperature. The analytical column was protected by a matching 10 × 3 mm drop-in guard column. The mobile phase consisted of 50/50/0.1 (v/v/v) ACN/H_2_O/Formic Acid and the flow rate was at 0.35 mL/min. To increase sample throughput, a paralleled SPE-HPLC configuration was developed with CTC HTS PAL autosampler equipped with trio valves. Figure 3 illustrates the flow paths for two independent SPE-HPLC streams. Paralleled analysis was achieved by offset dual stream system with a time delay, allowing efficient staggering of the MS acquisition time. Timing and triggering for injections, analytical pumps, loading/washing pumps and data collection were controlled by Cycle Composer software. Figure 4 shows a timing diagram for operating staggered dual stream SPE-HPLC analysis.

### 2.7. Mass spectrometry

A Perkin-Elmer (PE) Sciex (Thornhill, ON, Canada) API 3000 triple quadrupole mass spectrometer equipped with a Turbo Ionspray source operating in negative ion mode was used in the multiple ion monitoring (MRM) of analyte and IS. The Ionspray voltage was 3000 V and the source temperature was at 200 ºC. The flow of turbo ionspray gas (N2) was optimized at 8 L/min. The nebulizer gas, collision activated dissociation (CAD) gas, and the curtain gas were set to 8, 10 and 4 (arbitrary units), respectively. Other compound-dependent parameters were optimized for analyte and the same settings were used for IS. Detailed settings for each compound are given in Table 1. The mass analyzers Q1 and Q3 were operated at unit mass resolution. A pause time of 5 ms were added between each scans to avoid potential “cross talks”.

**Table 1:** Tuning Parameters for Analyte and IS.

Analyte	MRM Transition	Dwell Time (ms)	Declustering Potential (V)	Collision Energy (V)	Collision Cell Exit Potential (V)
ABT-925	*m/z* 457.4 → 329.4	400	40	32	15
d8-ABT-925 (IS)	*m/z* 465.5 → 337.5	400	40	32	15

Peak areas of the analyte and internal standard were integrated using Applied Biosystems/MDS Sciex Analyst^TM^ software. The integration area data were imported into Watson^®^ LIMS for regression and quantitation using internal standardization. A calibration curve was then derived from the peak area ratio *versus* the concentration of the standards. A weighting of 1/x^2^ (where x is the concentration of a given standard) was employed for curve fitting. The regression equation for the calibration curve was then used to back-calculate the measured concentrations. For each standard and quality control (QC) sample, the results were compared to the theoretical concentrations to obtain the accuracy of each level measured.

### 2.8. Method validation

The method was validated in human plasma (with potassium EDTA as anti-coagulant) according to the FDA guidelines and internal SOPs.

Three consecutive validation runs were designated to evaluate assay linearity as well as accuracy and precision. Each run included a reference solution sample for system verification, a blank sample, a blank sample with internal standard (IS), a set of calibration standards analyzed in singlet, six replicates of the lower limit of quantitation (LLOQ) evaluation sample, six replicates of the upper limit of quantitation (ULOQ) evaluation sample and six replicates of each QC. ULOQ and LLOQ evaluation samples were the highest and the lowest calibration standards, respectively. They were treated as QCs and not used in generating the calibration curve. The acceptance criteria for accuracy (expressed as %Bias) and precision (expressed as %CV) were set to be within 15%, except for LLOQ that was set at 20%. At least 75% of all calibration standards, two-thirds of LLOQ evaluation samples, two-thirds of ULOQ evaluation samples, half of each level of QCs and two-thirds of all QCs in a run must meet accuracy acceptance criteria. All replicated analysis at one concentration level must meet precision acceptance criteria. 

To demonstrate the selectivity of this method, 6 lots of matrix with and without IS were screened for interference from endogenous matrix components. Peak areas from the blank samples extracted without IS were compared with LLOQ peak area. At least 90% of the tested lots must have peak areas less than 20% of the LLOQ. All blank samples with extracted with IS must quantitate below the lower quantitation limit (BQL). 

To demonstrate that the assay performance is independent from the sample matrix, low QC samples were prepared using 6 different lots of matrix. Matrix effect QCs were analyzed in six replicates. At least 90% of all tested lots must have their mean concentration within 15% of the theoretical value. 

Recovery was accessed by comparing the peak area of recovery evaluation (RE) sample to that of recovery control (RC) solution. The RE samples were prepared in human plasma at three concentration levels. They were analyzed following the regular sample extraction process. The RC solutions were prepared in appropriate solvent at the same concentrations of the corresponding RE samples. They were injected directly onto HPLC column by passing the on-line SPE column. The recoveries for both analyte and IS were evaluated. 

### 2.9. Application of the method

The validated method was used in production to analyze two Phase II clinical studies. Individual incurred samples, accounting for approximately 10% of all samples in this study, were reassayed in singlet to evaluate the assay reproducibility. The values generated from the reassays were used to assess assay reproducibility, and were not used in pharmacokinetic calculations. The initial and the reassay values were compared.

## 3. Results and Discussion

### 3.1. Method development

Aqueous loading solutions at different pH values were evaluated for maximum loading efficiently by measuring analyte recovery.

During method development, different elution modes (forward elution versus backward elution) were investigated. When performing backward elution (Figure 3), peak shapes were generally sharper and the carryover caused by the residue analyte trapped in the extraction column was less. Therefore, backward elution mode was used.

System carryover was a particular concern when developing assays utilizing on-line SPE methodology. Different aqueous and organic wash solutions were evaluated to minimize system carryover. A strong base (0.1% Ammonium Hydroxide) wash has been proven to be very effective when using a polymer based extraction column.

In order to simplify sample preparation procedure, a supernatant transfer step was omitted after protein precipitation. Therefore, injection needle penetration depth into the 96-well plate was carefully set to avoid the injection of precipitated particulates. 

### 3.2. Method validation

#### 3.2.1. Selectivity

**Figure 5:**
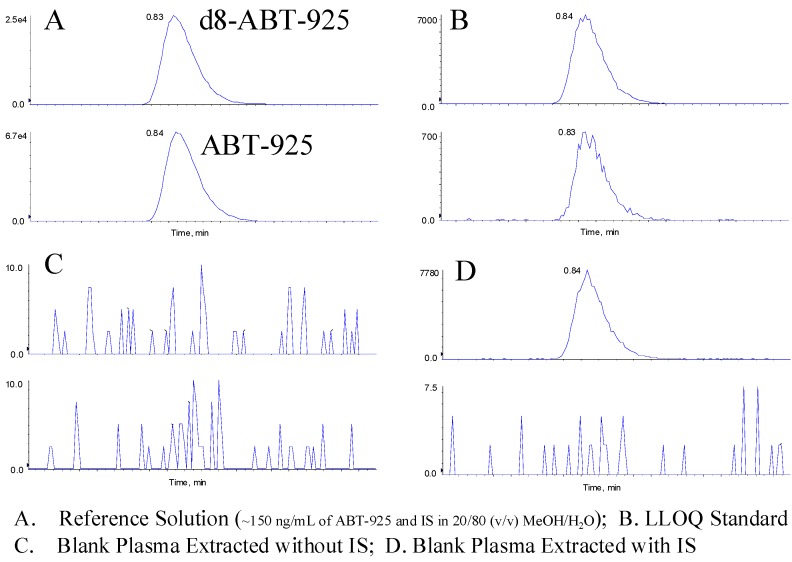
Representative Chromatograms

To demonstrate the selectivity of this method, six lots of normal human plasma were screened for interference from endogenous matrix components with and without the IS. All blank samples with IS quantitate BQL. For blank samples extracted without IS, no peaks above the threshold of peak detection were observed at the retention time of the analyte. Representative chromatograms of blank plasma with and without IS are shown in Figure 5.

#### 3.2.2. Linearity

The assay was linear over a concentration range of 0.0102 to 5.24 µg/mL. Using linear regression and a weighing factor of the inverse squared concentration (1/x^2^), correlation coefficient of 0.999124 or higher was obtained for the three consecutive accuracy and precision runs. Mean bias at lower limit of quantitation (LLOQ) was 1.7%. Mean Bias was between -2.7% and 1.1% for none LLOQ standards. 

#### 3.2.3. Accuracy and precision

Accuracy and precision of the assay were determined by replicate analysis (n = 6) of QC samples at four concentration levels. Accuracy and precision at both lower limit of quantitation (LLOQ) and upper limit of quantitation (ULOQ) were also evaluated by replicate analysis (n = 6) of LLOQ and ULOQ standards as QCs, respectively. Statistics were calculated for both intra-run and inter-runs for all runs designated for the evaluation of assay accuracy and precision. The results are summarized in Table 2.

**Table 2:** Statistical Summary of the Assay Accuracy and Precision.

	Run Number	ULOQ	QC1	QC2	QC3	QC4	LLOQ
		5.24 (μg/mL)	4.16 (μg/mL)	0.999 (μg/mL)	0.160 (μg/mL)	0.0256 (μg/mL)	0.0102 (μg/mL)
Intrarun Mean	1	5.32	4.18	0.988	0.157	0.0250	0.0103
Intrarun SD		0.0383	0.0327	0.00703	0.00295	0.000650	0.000262
Intrarun %CV		0.7	0.8	0.7	1.9	2.6	2.5
Intrarun %Bias		1.6	0.4	-1.1	-1.8	-2.3	0.7
n		6	6	6	6	6	6
Intrarun Mean	2	5.29	4.18	0.999	0.158	0.0255	0.0108
Intrarun SD		0.0634	0.0505	0.00737	0.00164	0.000373	0.000266
Intrarun %CV		1.2	1.2	0.7	1.0	1.5	2.5
Intrarun %Bias		1.0	0.4	0.0	-1.2	-0.3	5.6
n		6	6	6	6	6	6
Intrarun Mean	3	5.29	4.27	0.995	0.158	0.0256	0.0101
Intrarun SD		0.0920	0.192	0.0331	0.00696	0.000742	0.000333
Intrarun %CV		1.7	4.5	3.3	4.4	2.9	3.3
Intrarun %Bias		1.0	2.6	-0.4	-1.2	0.1	-1.2
n		6	6	6	6	6	6
Mean Concentration Found		5.30	4.21	0.994	0.157	0.0253	0.0104
Inter-run SD		0.0651	0.118	0.0193	0.00424	0.000631	0.000401
Inter-run %CV		1.2	2.8	1.9	2.7	2.5	3.9
Inter-run %Bias		1.2	1.1	-0.5	-1.8	-1.1	1.7
n		18	18	18	18	18	18

#### 3.2.4. Matrix effect

To demonstrate that the assay performance is independent from the sample matrix, low QC samples were prepared using six different lots of matrix. Accuracy and precision of matrix effect QCs are tabulated in Table 3. Consistent analytical recovery was achieved for all the lots tested. The mean bias from each lot ranged from -3.6% to 0.7%.

**Table 3:** Statistical Summary of the Precision and Accuracy of Matrix Effect Evaluation Samples.

	MEQC_Lot 1	MEQC_Lot 2	MEQC_Lot 3	MEQC_Lot 4	MEQC_Lot 5	MEQC_Lot 6
	0.0231 (μg/mL)	0.0231 (μg/mL)	0.0231 (μg/mL)	0.0231 (μg/mL)	0.0231 (μg/mL)	0.0231 (μg/mL)
Replicate #1	0.0238	0.0228	0.0234	0.0252	0.0244	0.0258
Replicate #2	0.0238	0.0234	0.0230	0.0225	0.0222	0.0246
Replicate #3	0.0241	0.0236	0.0242	0.0235	0.0230	0.0224
Replicate #4	0.0221	0.0220	0.0219	0.0229	0.0233	0.0230
Replicate #5	0.0224	0.0216	0.0221	0.0225	0.0214	0.0226
Replicate #6	0.0220	0.0206	0.0221	0.0221	0.0226	0.0213
Mean	0.0230	0.0223	0.0228	0.0231	0.0228	0.0233
SD	0.000965	0.00115	0.000911	0.00113	0.00102	0.00163
%CV	4.2	5.2	4.0	4.9	4.5	7.0
%Bias	-0.6	-3.6	-1.4	-0.1	-1.4	0.7
n	6	6	6	6	6	6

#### 3.2.5. Recovery

Recovery control (RC) solutions were prepared in ACN. Recovery evaluation (RE) samples were prepared in human plasma. 50 µL RC solutions were aliquoted into assay plate and fortified with 250 µL of IS. Wells containing RC solutions were mixed and dried down under room temperature N_2_. The dried wells were then reconstituted with 300 µL of 20/80 (v/v) MeOH/water. Reconstituted RC solutions were directly injected onto HPLC analytical column, bypassing extraction column. RE samples were aliquoted into assay plate following normal sample preparation procedure. RE samples were analyzed using the full extraction procedure with extraction column. The analyte was tested at three concentration levels, and the IS was tested at one concentration level. Mean extraction recoveries for analyte and IS are 81.6% and 73.0%, respectively.

#### 3.2.6. Stability

The storage stability of ABT-925 in human plasma (Potassium EDTA as anticoagulant) was evaluated to determine the degradation occurred during long-term frozen storage. ABT-925 is stable for at least 604 days when stored frozen at -20 ºC. Freeze-thaw and short- term room temperature stability were also evaluated. Evaluation QC samples were thawed completely unassisted, held at room temperature for a documented period of time and returned to the -20 ºC freezer for storage. The concentration of the evaluation samples was computed using the calibration curve and compared to those QCs that underwent only one freeze-thaw cycle. No apparent potency loss was found after five simulated sampling cycles and approximately 34.5 h at room temperature.

The stabilities of aliquoted samples stored in an autosampler or a refrigerator were also evaluated. A prepared batch is stable for at least 69 h in a cooled autosampler (set point at 10 ºC), and it is stable for at least 74.5 h in a refrigerator (temperature between 2 to 8 ºC). 

#### 3.2.7. System carryover evaluation

One challenge associated with on-line SPE methodology has always been the system carryover. In certain cases, the assay dynamic range may need to be reduced in order to meet the criteria for carryover evaluations. The main contributors to system carryover are autosampler, extraction column and all the components on the complicated fluid path for on-line SPE. We have identified the main source of system carryover in this assay is the extraction column. Different organic and aqueous wash solutions were tested to minimize system carryover. During validation, blanks with IS were injected after ULOQs. From six replicate tests, the mean peak area of the blanks accounted for less than 14% of the mean peak area of the LLOQ. 

### 3.3. Method application

The validated method was applied to analyze the following two Phase II clinical studies:

- Randomized, Placebo-controlled Study to Evaluate the Safety and Efficacy of ABT-925 in Subjects with Acute Exacerbation of Schizophrenia
- Open-Label Positron Emission Tomography (PET) Study to Evaluate the Effects of Single and Multiple Doses of ABT-925 on [11C]-(+)-PHNO Binding Potential to D3 Receptors in the Brain of Healthy Subjects

Incurred sample reanalysis was performed in the PET study. Approximately 10% of total samples analyzed were repeated in singlet. Two samples were selected from each subject. One is at concentration approximate the Cmax level. The other is in the elimination phase, approximately three times of the assay LLOQ concentration. %Difference was calculated using (Repeat Value – Original Value)/Mean × 100. The %Difference ranges from -11.2 to 7.0 with a mean at -3.6. 

## 4. Conclusions

A sensitive, specific and reproducible method was developed and utilized for the analysis of ABT-925 in human plasma. The implementation of on-line SPE sample cleanup has provided clean sample extracts that allowed high throughput sample analysis without interruption of detector maintenance. The simple sample processing procedure, along with the automation, made the method robust and less error prone.
